# Correlation-based common spatial pattern (CCSP): A novel extension of CSP for classification of motor imagery signal

**DOI:** 10.1371/journal.pone.0248511

**Published:** 2021-03-31

**Authors:** Khatereh Darvish ghanbar, Tohid Yousefi Rezaii, Ali Farzamnia, Ismail Saad

**Affiliations:** 1 Department of Biomedical Engineering, University of Tabriz, Tabriz, East Azarbijan, Iran; 2 Faculty of Engineering, Universiti Malaysia Sabah, Kota Kinabalu, Sabah, Malaysia; Torrens University Australia, AUSTRALIA

## Abstract

Common spatial pattern (CSP) is shown to be an effective pre-processing algorithm in order to discriminate different classes of motor-based EEG signals by obtaining suitable spatial filters. The performance of these filters can be improved by regularized CSP, in which available prior information is added in terms of regularization terms into the objective function of conventional CSP. Variety of prior information can be used in this way. In this paper, we used time correlation between different classes of EEG signal as the prior information, which is clarified similarity between different classes of signal for regularizing CSP. Furthermore, the proposed objective function can be easily extended to more than two-class problems. We used three different standard datasets to evaluate the performance of the proposed method. Correlation-based CSP (CCSP) outperformed original CSP as well as the existing regularized CSP, Principle Component Cnalysis (PCA) and Fisher Discriminate Analysis (FDA) in both two-class and multi-class scenarios. The simulation results showed that the proposed method outperformed conventional CSP by 6.9% in 2-class and 2.23% in multi-class problem in term of mean classification accuracy.

## Introduction

One of the most popular topics in computer science in the last decade has been research on Brain-Computer Interface (BCI) systems. BCI systems consist of sensors and a signal processing unit that can convert brain activities into logical commands for an external device or computer application. The basis of these systems is decoding brain signals using feature extraction and pattern recognition algorithms [1, 2].

Motor imagery (MI) is a dynamic state in the brain that a subject imagines a body movement such as hand or tongue movements without any external movement or muscle activation [3]. This imagination task leads to neural activities in the primary sensorimotor cortex so similar to real movement which has different pattern depends on the type of imagination [4]. MI based-BCI systems have designed to detect different MI signals that can be helpful for people with motor disabilities or mental injuries to do some activities such as moving an artificial arm, spelling words or controlling a computer cursor [1, 5].

One of the useful and powerful pre-processing methods for feature extraction in BCI is Common Spatial Pattern (CSP). CSP approach was used for the first time in detection of EEG abnormality [6]. Then, CSP filters applied to the multi-channel EEG signal for the classification of left and right hand MI signals [7]. Initially, CSP algorithm finds spatial filters for two classes of data. Through applying spatial filters to the data, the variance is maximized for one class of data while it is minimized for the other one. Therefore, two classes have more distinctive feature to be used in the classification stage.

Despite the mentioned advantages for the CSP method, this approach has such shortcomings as noise sensitivity, overfitting and two-class issue, which has led quite a few studies to propose extensions for it [5, 8–10].

CSP approach uses L2 norm in its optimization problem; as a consequence, it has noise sensitivity problem. In other words, outlier data and artifacts maybe have exaggerated effect as they mostly have large amplitude in brain signals. Therefore, L1 norm has been proposed based on CSP for solving this problem in [11]. This work used L1-norm method in the objective function instead of L2 norm to find eigenvectors. In the other work, Sparse CSP-L1 (sp-CSPL1) used L1 norm technique to solve optimization problem of CSP in two steps. The first one finds the robust spatial filters, and the other one penalizes the objective function by adding a penalty term to induce sparsity [12]. Correntropy Induced Metric based CSP (CSP-CIM) has been proposed to obtain a robust algorithm against outliers in [13]. CIM is used instead of L2 norm to approximate the objective function of CSP in the different dynamic regions. Another way to reduce the effect of artifacts and outlier data in obtaining spatial filters has been suggested in [14]. In this approach artifacts of data are removed in the preprocessing stage; therefore covariance matrices are calculated more accurate, as result CSP filters improved. As we know, an EEG signal includes amplitude and phase information; however, the phase information entirely ignored in CSP filter calculation. To solve this problem, three different methods have been presented in [15]. In the first method, phase information of EEG signal added to CSP objective function. In the second method, the CSP filters obtained by adding both amplitude and phase information to the objective function. Finally, the third approach has been used to handle nonlinearity in complex data points by the non-linear counterpart of Principal Component Analysis (NLPCA).

Non-stationarity of EEG signal occur for several reasons such as alternation in cognitive state of subject arising from mental fatigue and etc. adaptive common spatial pattern (ACSP) has been used to modify CSP filters based on mental fatigue of subject. The Results show ACSP outperformed conventional CSP in the term of class separability [16]. Regularized CSP (RCSP) has been proposed for solving noise sensitivity and non-stationarity problem in [17]. In this paper, CSP filters have been optimized by prior information about the noise of data. The other way to regularize the standard CSP algorithm is adding prior information to the covariance matrix estimation. Four new RCSP algorithms, including two proposed regularization terms for optimizing the objective function, are suggested in [18]. As a result, their proposed Tikhonov Regularization CSP (TRCSP) and Weighted Tikhonov Regularized CSP (WTRCSP) performed better than the CSP and other ones. Both of these approaches obtain spatial filters by adding prior information to the objective function. CSP approach can only extract spatial information of the brain activities. Regularizing Multi-bands CSP (RMCSP) has been proposed that extracts data in spectral, temporal and spatial domains using Tikhonov regularization method in [19]. CSP algorithm originally proposed to recognize between two classes of data. CSP depends on frequency bands that it is different for each subject regarding to individual characteristic nevertheless these differences have been neglected. Also CSP has sample based covariance matrix. To overcome these problems, a feature extraction approach has been suggested based on filter bank method in [20].

Turning to the two-class problem, as CSP was originally proposed to discriminate between two classes of data, first time Pair wise approach for multi-class problems has been proposed in [21]. Furthermore, One-Versus-the-Rest (OVR) approach has been proposed to tackle with multi-class problems [22], which cannot be considered as a genuine extension of two-class CSP to more than two-class cases. In [23] a new classification method has been proposed based on fuzzy system for multi-class problem. The proposed method outperformed in comparison with existing classifiers such as Linear Discriminant Analysis (LDA) and Support Vector Machine (SVM).

Correlation is a statistical criterion to measure similarity between two different signals. As well as one of the useful methods for detection of task related activation in the brain is the correlation based methods [24]. According to this fact, Local Temporal Correlation Common Spatial Pattern (LTCCSP) has been proposed to obtain optimized spatial filters. Covariance matric estimation is a noise sensitive step in obtaining CSP filters which is improved by imposing local temporal correlation information into it [25]. In other work, a Correlation-based Channel Selection (CCS) method was introduced for MI-based BCI. In this algorithms the channels with high correlation coefficient selected to obtain spatial filters by using new RCSP method [26]. In all the correlation-based approaches correlation calculated in the same class and correlation between different classes is neglected. In the other hand MI signals are similar to each other especially right hand and left hand MI signals. So these matters led us to propose novel CSP method by adding correlation between different classes in the calculation of CSP filters. As mentioned, one of the methods to improve spatial filters is regularized CSP by adding a suitable penalty term to the objective function. Temporal correlation between EEG channels is a good criterion to measure the similarity of different channels (right hand and left-hand movement in particular). Due to this fact, regularized CSP can be done using temporal correlation between different classes of EEG signals as prior information. In this paper, a new regularized CSP (CCSP) based on temporal correlation is proposed to improve spatial filters in two-class scenario and it is further generalized to multi-class problem. Imposing the temporal correlation as penalty term in solving objective function to obtain the spatial filters is shown to be more effective than the existing methods in terms of discriminating the class-data, leading to higher classification accuracy. In this work, our focus is on obtaining optimized spatial filters using the proposed method for MI signals and all the simulation steps are done using this kind of signals as inputs. It should be noted that the primary results of this paper have been published in a conference paper [27].

The rest of this paper is organized as follows: First, we will provide the basis of the existing CSP and RCSP methods are Section 2. Then the proposed regularized multi-class CSP method will be given in Section 3. Furthermore, the block diagram of the proposed method, classification, feature extraction, and data description is also presented in Section 3. In Section 4, the simulation result of the proposed method is given and compared to the existing methods. Finally, concluding remarks are presented in Section 5.

## Background

In the following, the mathematical foundations of standard CSP as well as RCSP are given.

### Common spatial patterns

As pointed out previously, CSP is one of the most popular approaches for feature extraction in BCI technology. CSP finds spatial filters such that the variance of the transformed data is maximized for one class while it is minimized for the other one [7]. Suppose **X**_1_ and **X**_2_ stand for the EEG signals for classes 1 and 2, respectively; where $X_{i}\in R^{n\times c}$, *n* and *c* represent time sample and number of EEG channels, correspondingly. The solution of the following objective function is the desired spatial filters.

$$\underset{w}{\arg\;\max}\;J(w)=\frac{w^{T}X_{1}^{T}X_{1}w}{w^{T}X_{2}^{T}X_{2}w}=\frac{w^{T}C_{1}w}{w^{T}C_{2}w},$$ (1)

**w** denotes the spatial filter, *T* is the transposition operator and **C**_1_ and **C**_2_ represent covariance matrices of **X**_1_ and **X**_2_ respectively. There are several methods for solving the maximization problem (1). Using the Lagrange multiplier method, the constrained problem (1) is converted to the following unconstrained problem:

$$L(\lambda ,w)=w^{T}C_{1}w-\lambda (w^{T}C_{2}w-1),$$ (2)

where *λ* is the Lagrange multiplier. Therefore, to calculate the spatial filter **w**, the derivative of *L* must be taken and set equal to zero with respect to **w**, that is:

$$\begin{array}{l} \frac{\delta L}{\delta w}=2w^{T}C_{1}-2\lambda w^{T}C_{2}=0\\ \;\Rightarrow C_{1}w=\lambda C_{2}w\\ \;\Rightarrow C_{2}^{-1}C_{1}w=\lambda w \end{array}$$ (3)

So, we have the standard eigenvalue problem. The solution to (1) are the eigenvectors of $M=C_{2}^{-1}C_{1}$, corresponding to its largest and lowest eigenvalues.

### Regularized CSP

RCSP has been proposed to overcome some shortcoming of CSP such as being sensitive to noise and artifacts, non-stationary and overfitting. In the following, one of most common ways of regularizing standard CSP is explained. For this purpose, some prior information from data is used as a regularization term in the objective function [18]. Therefore the objective function (1) is modified as follows:

$$\underset{w}{\arg\;\max}\;J_{P_{1}}(w)=\frac{w^{T}C_{1}w}{w^{T}C_{2}w+\alpha Q(w)}$$ (4)

Where *α* denotes the regularization parameter (*α*≥0) and **Q**(**w**) is the penalty term (prior information). **Q**(**w**) can be defined in the form of non-quadratic or quadratic function. Considering a quadratic function as penalty term, i.e., **Q**(**w**) = **w**^*T*^**Kw**, where **K** denotes the prior information from the signal; the solution of (4), following the same Lagrangian approach gives:

$$L_{P_{1}}(\lambda ,w)=w^{T}C_{1}w-\lambda (w^{T}(C_{2}+\alpha Κ)w-1)$$ (5)

Taking the derivative with respect to **w** and setting it equal to zero, we have:

$$\begin{array}{l} \frac{\delta L}{\delta w}=2w^{T}C_{1}-2\lambda w^{T}(C_{2}+\alpha K)=0\\ \;\Rightarrow C_{1}w=\lambda (C_{2}+\alpha K)w\\ \;\Rightarrow {\left(C_{2}+\alpha K\right)}^{-1}C_{1}w=\lambda w \end{array}$$ (6)

The eigenvectors of **M**_1_ = (**C**_2_+*α***K**)^−1^**C**_1_ corresponding to its largest eigenvalues are the desired spatial filters. Also, to calculate the optimized CSP filters, the objective function $J_{P_{2}}(w)$ should be solved in the same manner.

$$\underset{w}{\arg\;\max}\;J_{P_{2}}(w)=\frac{w^{T}C_{2}w}{w^{T}C_{1}w+\alpha Q(w)}$$ (7)

The obtained filters by solving maximization problem (7) are the eigenvectors corresponding the largest eigenvalues of **M**_2_ = (**C**_1_+*α***K**)^−1^**C**_2_. Thus, the spatial filters are obtained by putting largest eigenvalue of **M**_1_ and **M**_2_ together. There are several penalty functions with the difference in **K** as prior information. The simple assumption is **K** = **I** which **I** represent identity matrix [18].

## Proposed Correlation-based CSP (CCSP) method

As mentioned before, CSP algorithm discriminates two classes of data by maximizing/minimizing their projected variance, which is initially designed for two classes of data. In order to overcome the shortcomings of the standard CSP and obtain a feature extraction method which can be easily generalized to multi-class cases, we proposed to exploit the temporal correlation between signals as a measure of similarity between them and impose it as penalty term to obtain a regularization CSP. In the following, at first the proposed CCSP algorithm is formulated for two-class problem. Then, its generalization for multi-class problem is given in the subsequent section.

### Two-class problem

In the previous section, the basis of the RCSP approach is presented. In this section, the formulation of the proposed method based on the temporal correlation as a penalty term for two-class problem is given. Consider the penalty term, **Q**_1_(**w**), to be a quadratic function of **w** as:

$$Q_{1}(w)=w^{T}R_{d1}w$$ (8)

where **Q**_1_(**w**) denotes the penalty term in $J_{P_{1}}(w)$ and $R_{d1}\in R^{c\times c}$ is the diagonal matrix in which the elements of the main diagonal are the correlation between ${\bar{X}}_{1}$ and ${\bar{X}}_{2}$. ${\bar{X}}_{1}$ and ${\bar{X}}_{2}$ represent the average of **X**_1_ and **X**_2_ for all of the trials, respectively; i.e. (in order to reduce computational complexity, ${\bar{X}}_{1}$ and ${\bar{X}}_{2}$ are used instead of **X**_1_ and **X**_2_)

$${\bar{X}}_{i}=\frac{\sum_{j=1}^{t}X_{ij}}{t},\;i=1,2$$ (9)

where *i* and *j* represent the class and trial indices, respectively, and *t* is the total number of trials for each class. Assume **R** to be the correlation matrix between two different classes as:

$$R=\left[\begin{array}{cccc} r_{11} & r_{12} & \cdots & r_{1c}\\ r_{21} & r_{22} & \cdots & r_{2c}\\ \vdots & & \ddots & \vdots \\ r_{c1} & r_{c2} & \cdots & r_{cc} \end{array}\right]$$ (10)

where $r_{ij}=\mathrm{corr}(\bar{x_{1}^{i}},\bar{x_{2}^{j}}),$ *i*,*j* = 1,2,…c.

$\bar{x_{1}^{i}}$ and $\bar{x_{2}^{j}}$ denote the *i*-th and *j*-th column of the matrices $\bar{X_{1}}$ and $\bar{X_{2}}$, respectively, and corr(.) is the correlation operator. Having the correlation matrix **R**, **R**_*d*1_ is constructed as follows:

$$R_{d1}=\left[\begin{array}{cccc} a_{1} & 0 & \cdots & 0\\ 0 & a_{2} & & 0\\ \vdots & & \ddots & \\ 0 & 0 & & a_{c} \end{array}\right]$$ (11)

where the diagonal entries, *a*_*i*_, are obtained as:

$$a_{i}=\frac{\sum_{j=1}^{c}\left|r_{ij}\right|}{c},\;i,j=1,2,\ldots ,c$$ (12)

and |.| denotes the absolute operator. Considering the penalty term for $J_{P_{2}}(w)$, i.e., **Q**_2_(**w**), it is also defined in the similar fashion as:

$$Q_{2}(w)=w^{T}R_{d2}w$$ (13)

where $R_{d2}\in R^{c\times c}$ is the diagonal matrix of the form:

$$R_{d2}=\left[\begin{array}{cccc} b_{1} & 0 & \cdots & 0\\ 0 & b_{2} & & 0\\ \vdots & & \ddots & \\ 0 & 0 & & b_{c} \end{array}\right]$$ (14)

and *b*_*j*_ are calculated as:

$$b_{j}=\frac{\sum_{i=1}^{c}\left|r_{ij}\right|}{c},\;i,j=1,2,\ldots ,c$$ (15)

The spatial filters are obtained by replacing the proposed penalty terms **Q**_1_(**w**) and **Q**_2_(**w**) in (4) and (7) and solving the resulting objective functions. In order to maximize $J_{P_{1}}(w)$ and $J_{P_{2}}(w)$, **Q**_1_(**w**) and **Q**_2_(**w**) must be minimized. Therefore, the EEG channels of higher correlation will have a lower contribution in solution of the optimization problem, which leads to having more discriminative projected signals.

Two clear the computational complexity concept that is mentioned before, assume *e* and *k* show the trials number of **X**_1_ and **X**_2_ respectively. Therefore the computational order to calculate the correlation between two different classes is *O*(e×*k*×*n*×*c*) that *n* and *c* represent time sample and number of EEG channels for **X**_*i*_. By using ${\bar{X}}_{1}$ and ${\bar{X}}_{2}$ instead of **X**_1_ and **X**_2_ the computational order of correlation matric is reduced to *O*(*n*×*c*). It must be noticed however the computational complexity is decreased but it is still high in comparison to conventional CSP.

### Multi-class problem

In the following, the proposed two-class CCSP will be extended to more than two class problems by defining a suitable objective function for the spatial filters of each class. Considering the OVR classification method, the spatial filters for one class are calculated against the others [8]. Likewise the two-class problem, the temporal correlations are used as penalty terms to the objective functions. That is, the temporal correlations between one class and the remaining classes are calculated. Then, sum of the calculated temporal correlations is used to impose these correlations to the corresponding objective function. Take for example a four-class problem. Using the above described approach we have the following objective functions for:

$$\underset{w}{\arg\;\max}\;J_{P_{1}}(w)=\frac{w^{T}C_{1}w}{w^{T}C_{2}w+w^{T}C_{3}w+w^{T}C_{4}w+\alpha Q_{1}(w)}$$ (16)

$$\underset{w}{\arg\;\max}\;J_{P_{2}}(w)=\frac{w^{T}C_{2}w+w^{T}C_{3}w+w^{T}C_{4}w}{w^{T}C_{1}w+\alpha Q_{2}(w)}$$ (17)

In which **Q**_1_(**w**) and **Q**_2_(**w**) are calculated as follows:

$$Q_{1}(w)=w^{T}(R_{d1}^{12}+R_{d1}^{13}+R_{d1}^{14})w=w^{T}R_{d1}w$$ (18)

$$Q_{2}(w)=w^{T}(R_{d2}^{12}+R_{d2}^{13}+R_{d2}^{14})w=w^{T}R_{d2}w$$ (19)

where $R_{d1}^{12}$, $R_{d1}^{13}$ and $R_{d1}^{14}$ represent diagonal correlation matrices that are calculated using correlation between class 1 and 2, class 1 and 3 and class 1and 4, respectively; entries of which are calculated using (12). Likewise, $R_{d2}^{12}$, $R_{d2}^{13}$ and $R_{d2}^{14}$ denote diagonal correlation matrices between classes 1 and 2, 1 and 3 and 1 and 4, respectively; entries of which are calculated using (15). The problem for class 1 against the other classes is calculated by replacing **Q**_1_(**w**) and **Q**_2_(**w**) in (16) and (17) and solve these functions. This procedure can be followed to obtain the spatial filters for the remaining classes.

### Block-diagram of the proposed MI signal classifier

**Fig 1:**
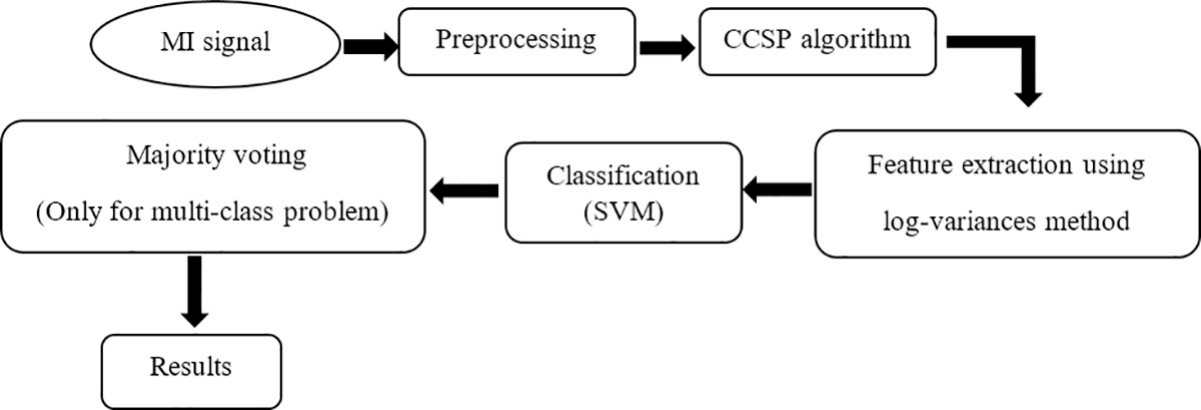
Block-diagram of the proposed method.

Fig 1 shows the block diagram of the proposed method. Pre-processing step in this block-diagram includes extracting different time duration from each trial of datasets and band-pass filtering. At first, time segments (it is explained in more details in the date description section) are extracted from each dataset. Then, each trial of data is band-pass filtered to make the mean of EEG signals equal to zero. For this purpose, each trial of EEG signals is passed through an 8–30 HZ band-pass filter (Butterworth filter of order 5). Then, CCSP method was applied to the filtered data to obtain the spatial filters. We used cross-validation to choose the best regularization parameter (*α*) from [10^−6^: 5×10^−5^: 10^−3^]. The next step is feature extraction, which is done using the log-variances method. In the fourth step, the extracted features are fed to the classifier. It should be noted that the majority voting step is used only for multi-class problem. Finally, the results are achieved.

### Classification and feature extraction

In this paper, log-variances method, a traditional and powerful method, was used to extract feature vector from the projected MI signals. As mentioned before CSP is a preprocessing method based on maximization the variance between different classes. But it is not a proper feature extraction method itself. So performing a method that uses variance as the feature can be helpful. By using log-variances method, we extract feature vectors with more class separability. Besides the data is projected into the normal distribution by using logarithm operator; therefore it can cause the ease of classification step [7, 28]. Assuming a 2*l*×*n* dimensional matrix as the filtered signal using 3 pairs of spatial filters (*l* = 3), the feature vector for each trial is calculated as:

$$f_{ij}=\log\frac{\mathrm{var}(z_{ij})}{\sum_{j=1}^{2l}\mathrm{var}(z_{ij})}$$ (20)

where *f*_*ij*_ denotes feature from *i*-th class and *j*-th filter and **z**_*ij*_ represents the projected signal of the *i*-th class and *j*-th filter. Furthermore, log(.) and var(.) denote the logarithm and variance operators, respectively.

There are various classification methods for classifying the MI signals which some of them are popular and more efficient than the others such as LDA, SVM and K-Nearest Neighbor (KNN). Also several extensions have been proposed for each of these classifiers to improve their performances.

LDA is a simple and effective classifier which is separated two or more classes of data by hyper-planes [29]. This classifier has been extensively used for classifying the MI signals that reduce the risk of misclassification but it may do not work properly when the testing data outnumbered training ones [30, 31].

As mentioned above KNN is one of the most common and powerful methods for classification of EEG signals. In this method, each data devote to the class number which is most common amongst its K nearest neighbour by a distance function such as Euclidean distance [29, 32]. Two parameters affect on the performance of KNN classifier: 1) value of K 2) distance function [32].

Another popular classifier for BCI applications is SVM which is a supervised learning method. This method separates two classes of data by finding the best hyper-plane which can create the largest distance from each class [33]. This method extended to classified multi-class data in [34]. Unlike LDA, SVM method represents a good performance in the cases that the number of features is high [33].

In this work, we have used SVM classifier for two and multi-class classification problems. It is an efficient and suitable method for classifying motor imagery signals. In two-class problem, feature vector is fed to the SVM classifier and the results are obtained but in multi-class problem after using classifier, output of the SVM classifiers are combined by the majority voting strategy and the final results are obtained as follows:

$$\omega =\underset{\omega =1,2,3,4}{\arg\;\max}\;p_{OVR}(\omega |x)$$ (21)

where *p*_*OVR*_(*ω*|x) denotes the probability of *x* to be classified as class 1, 2, 3 or 4.

It should be pointed out 5-fold cross-validation is used to choose the best hyper-plane in the classification step.

### Data description

In this study, three publically available datasets are used for simulation and comparison between the performance of the proposed algorithm and the existing ones. Each subject performed imagery movement and EEG signals of each subject have been recorded by several electrodes. These datasets consist of 17 subjects in overall. The EEG signal of 12 subjects have been used to simulate multi-class problem (dataset IIIa, BCI competition III and dataset IIa, BCI competition IV), while all of them have been used for two- class problem. The details of each dataset are given below:

1. Dataset IIIa, BCI competition III: In this dataset [35], 3 subjects participated in recording EEG signals. Four motor imagery tasks have been performed by them namely the imagination of the right hand, left hand, foot and tongue. The dataset was recorded using 60 EEG channels at 250 Hz sampling rate. There are 45 trials per class for training and 45 trials per class for testing for subject 1. Also, there is 30 trial per class for training and 30 trial per class for testing for each of the subject 2 and 3. We have used from right hand and left hand trials for two-class problem and all of the trials in multi-class problem.
2. Dataset IVa, BCI competition III: This dataset [35] consists of three motor imagery movements for 5 subjects including right hand, left hand and right foot, although only right hand and right foot signals are provided in BCI competition datasets. Signal are sampled with 100 Hz and 118 electrodes have been used to record EEG signals. 280 trial are recorded for each subject that 168, 224, 84, 56 and 28 trials were used for training set and rest of them for testing set.
3. Dataset IIa, BCI competition IV: This dataset [36] consists of EEG signals from 9 subjects for four motor imagery tasks (right hand, left hand, both feet and tongue). These data were recorded using 25 electrodes that 3 of them were recording EOG signals. In this paper, only EEG signals were used. There are 72 trials per class for training set and 72 trial per class for testing set. The sampling rate is 250 Hz. Right hand and left hand imagination movement have been used for simulating two-class problem.

Each dataset has particular settings; therefore, different time segment was extracted from each of the datasets for training and testing purposes [19]. In dataset IIIa, BCI competition III, time segments of duration 3.5-7s are extracted from each trial. This time range is 0–3.5s (whole time interval) for dataset IVa, BCI competition III. Finally, time duration of 2.5-6s was used for dataset IIa, BCI competition IV.

## Simulation results

**Fig 2:**
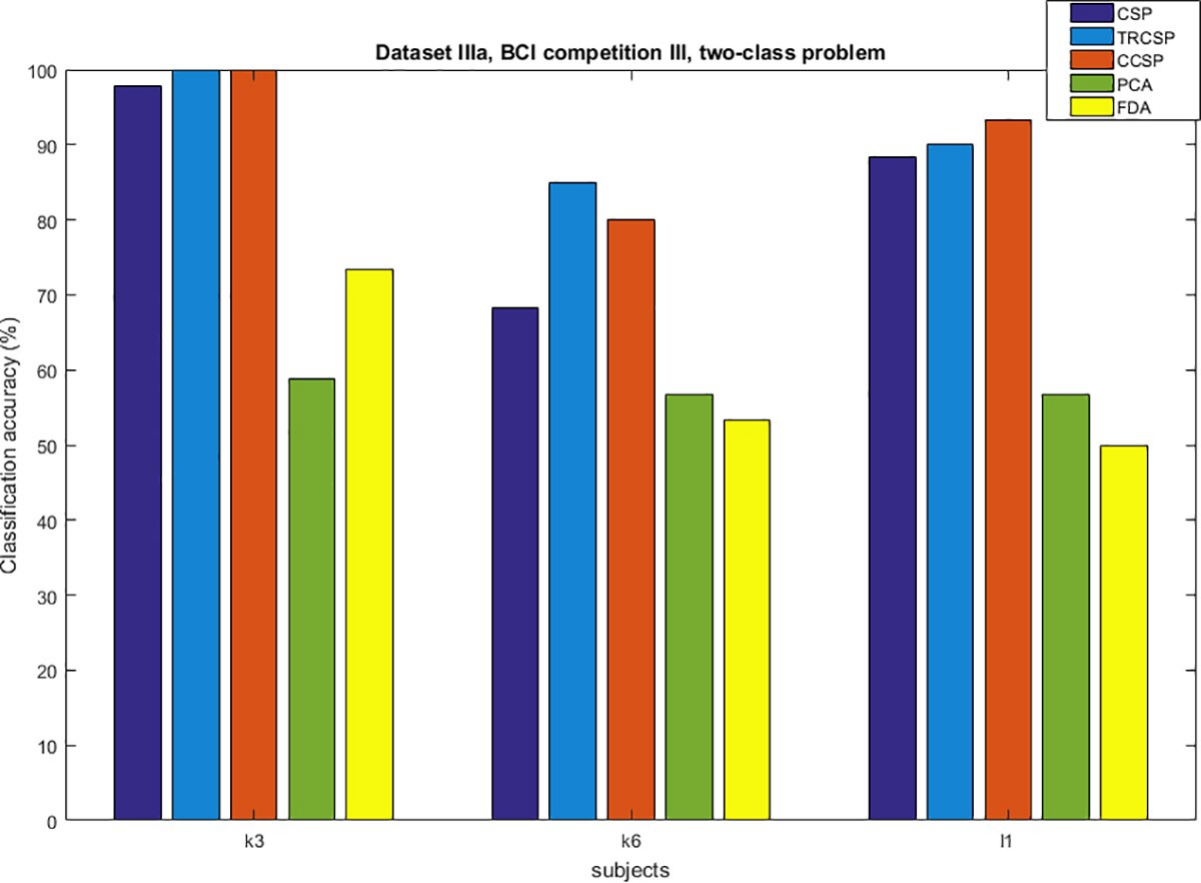
Comparison between classification accuracy of methods for two-class problem in dataset IIIa, BCI competition III.

**Fig 3:**
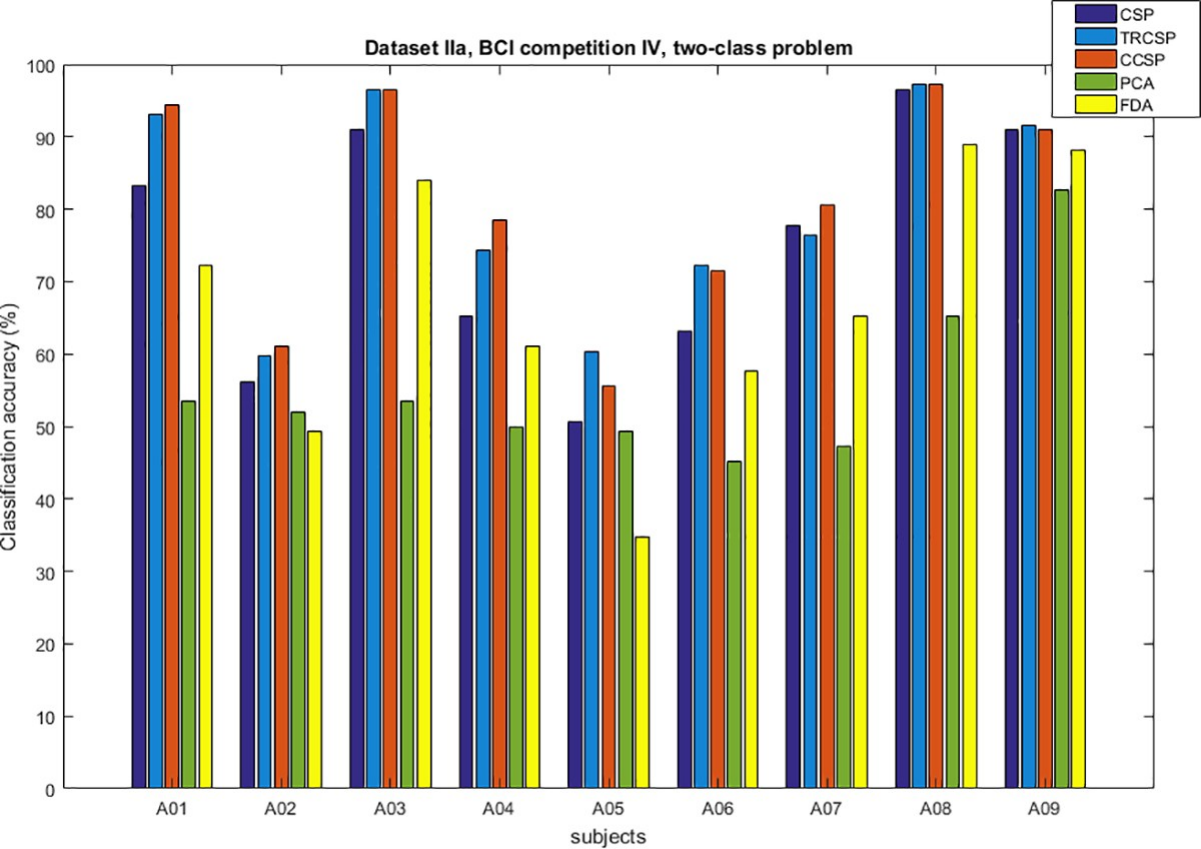
Comparison between classification accuracy of methods for two-class problem in dataset IIa, BCI competition IV.

**Fig 4:**
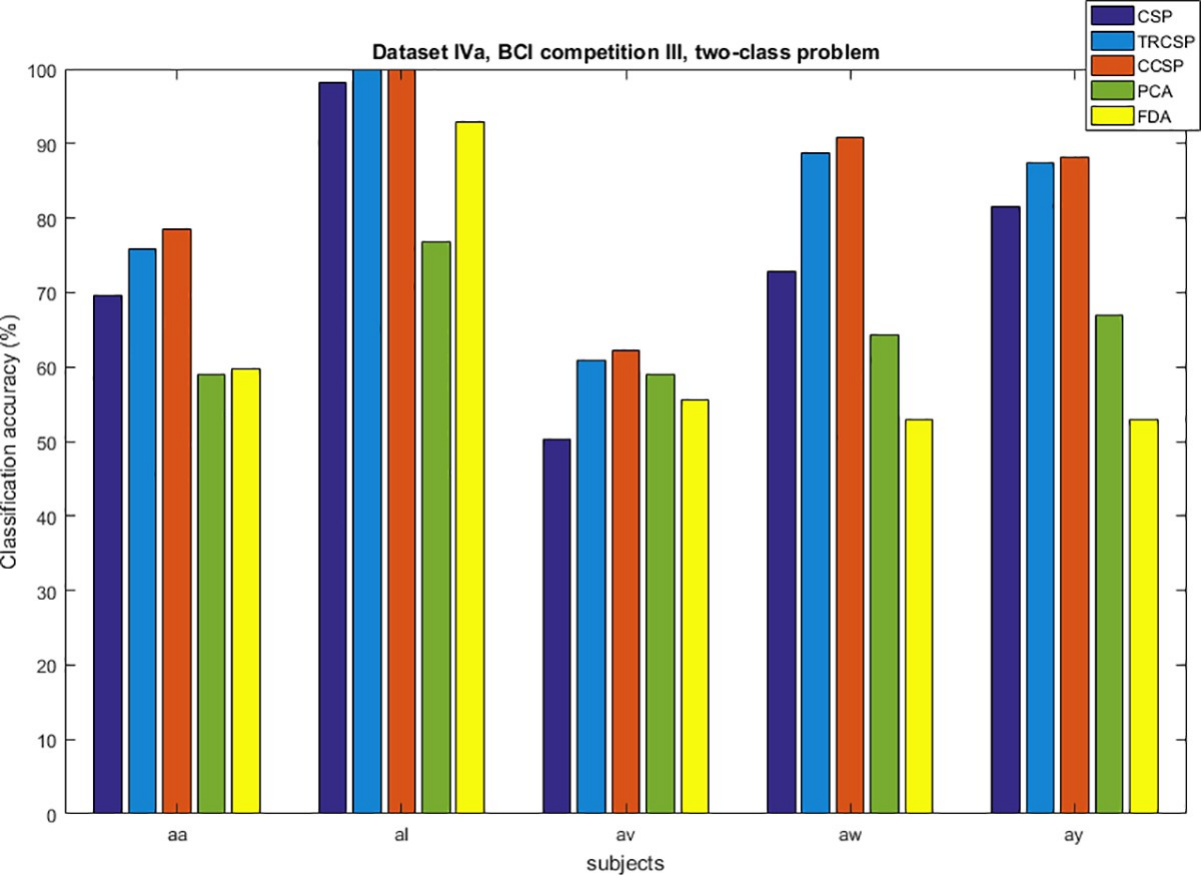
Comparison between classification accuracy of methods for two-class problem in dataset IVa, BCI competition III.

**Fig 5:**
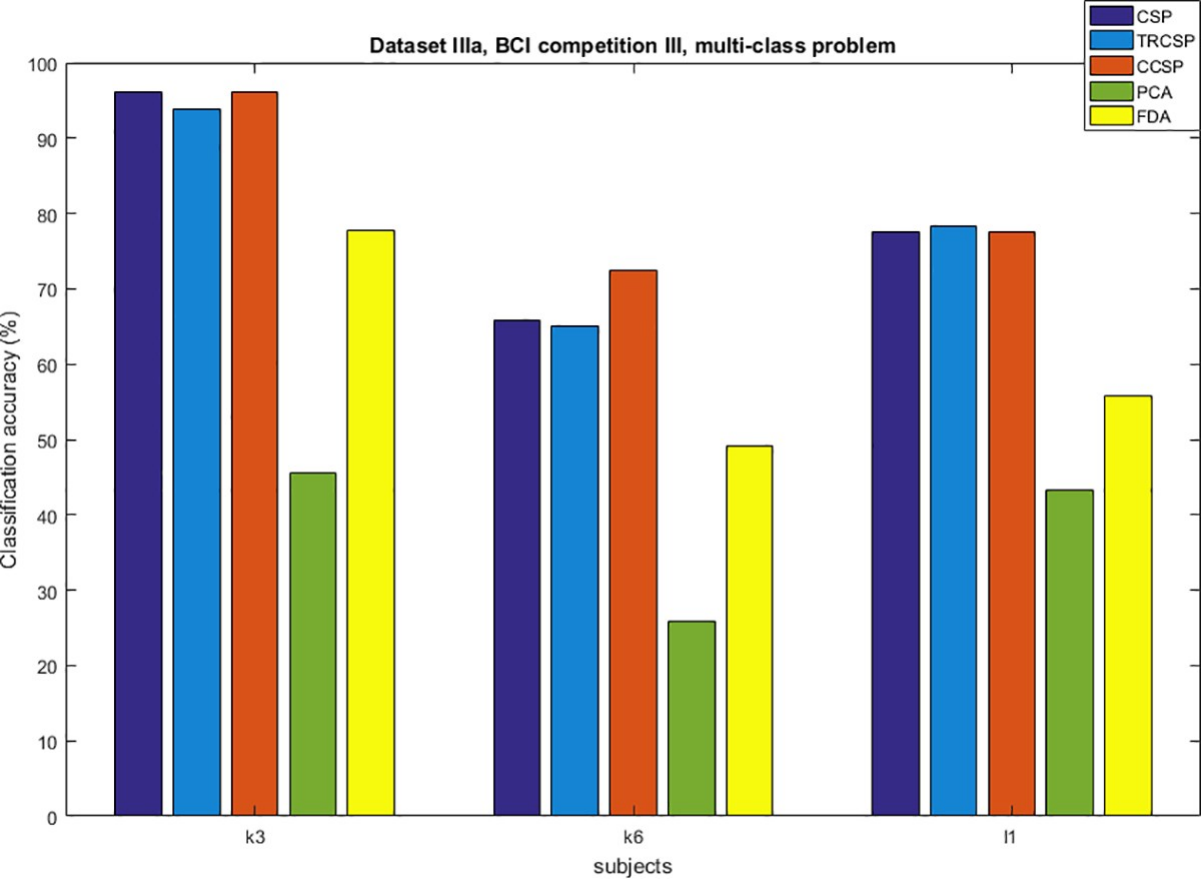
Comparison between classification accuracy of methods for multi-class problem in dataset IIIa, BCI competition III.

**Fig 6:**
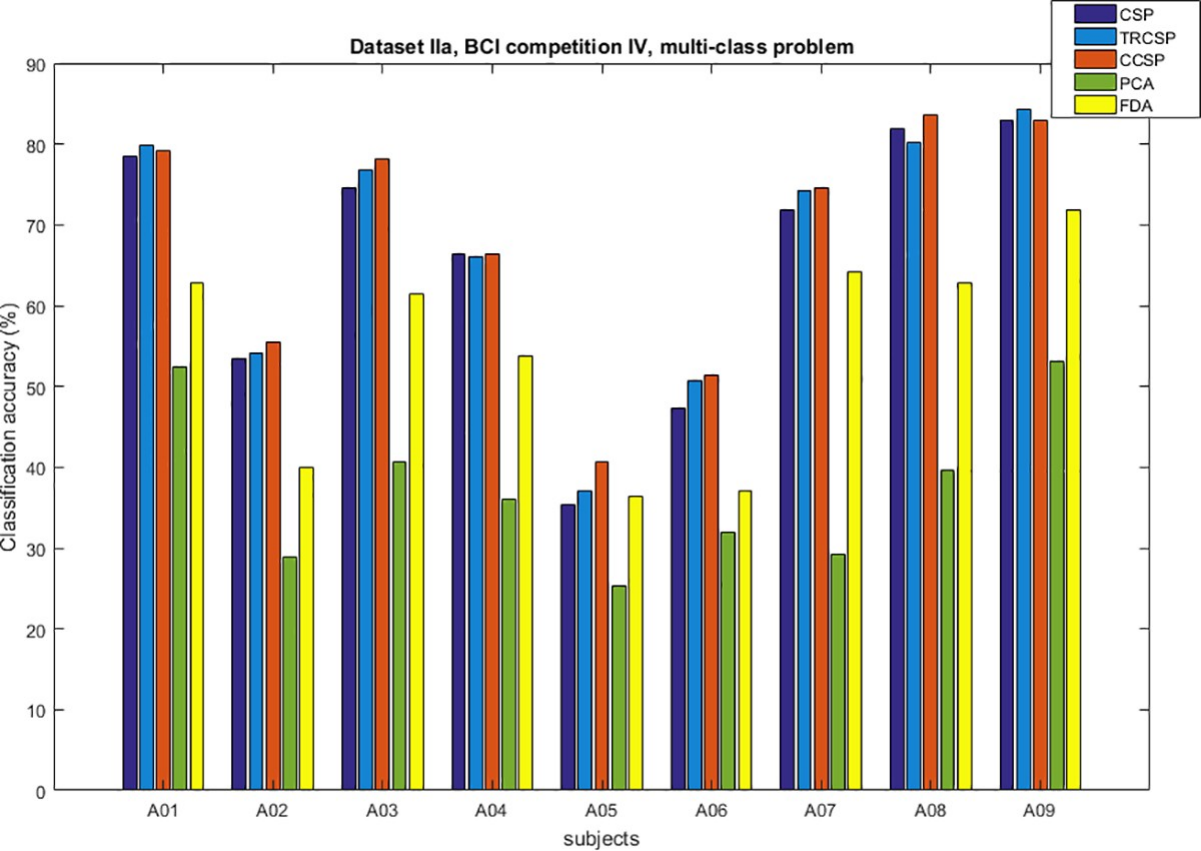
Comparison between classification accuracy of methods for multi-class problem in dataset IIa, BCI competition IV.

**Fig 7:**
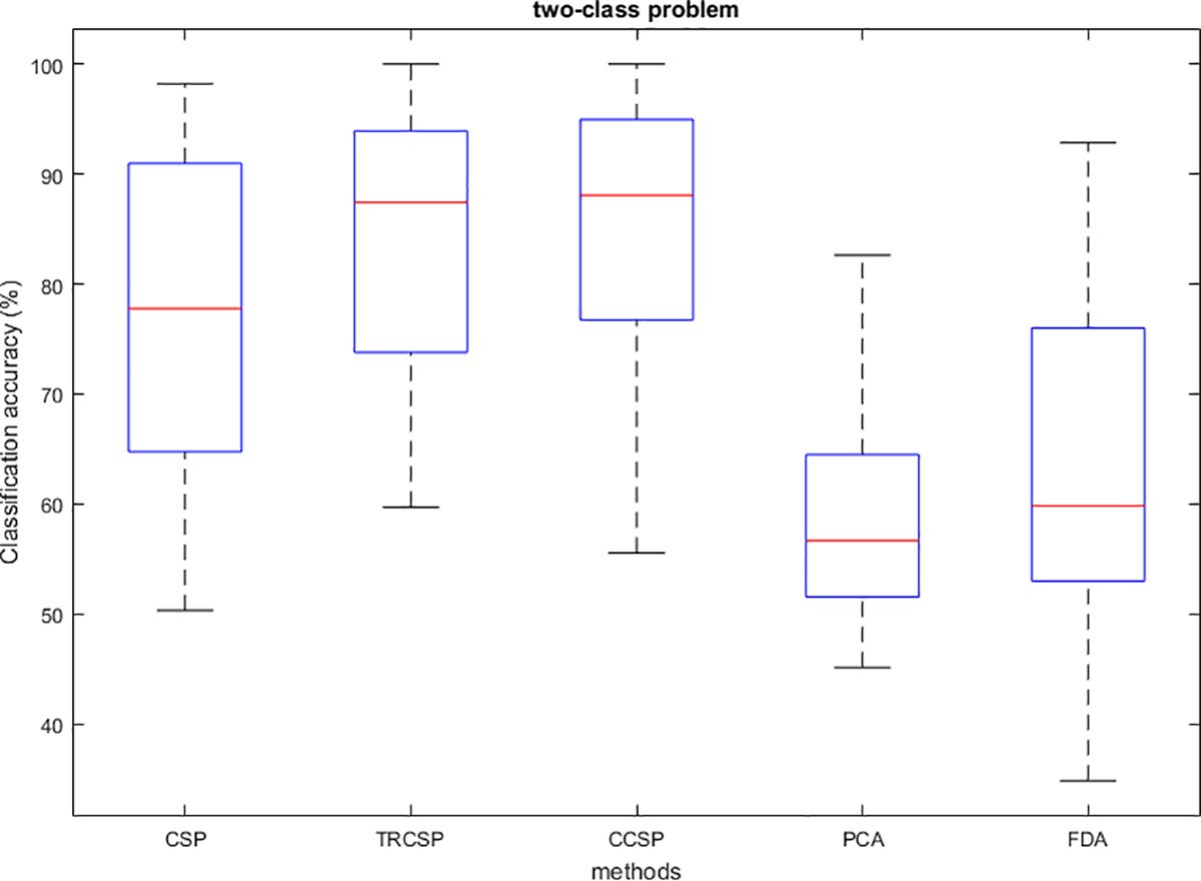
Boxplot of all subject for CSP, TRCSP and CCSP in two-class problem.

**Fig 8:**
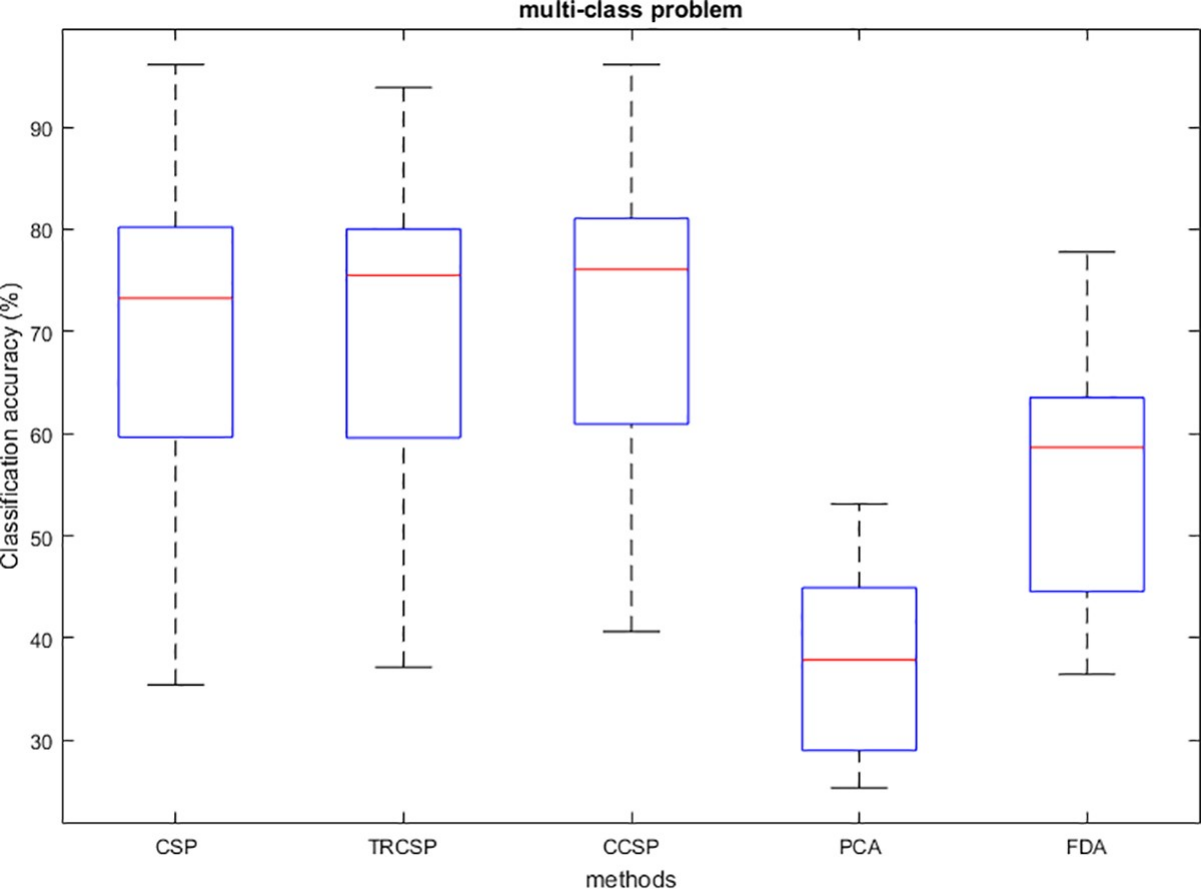
Boxplot of all subject for CSP, TRCSP and CCSP in multi-class problem.

**Fig 9:**
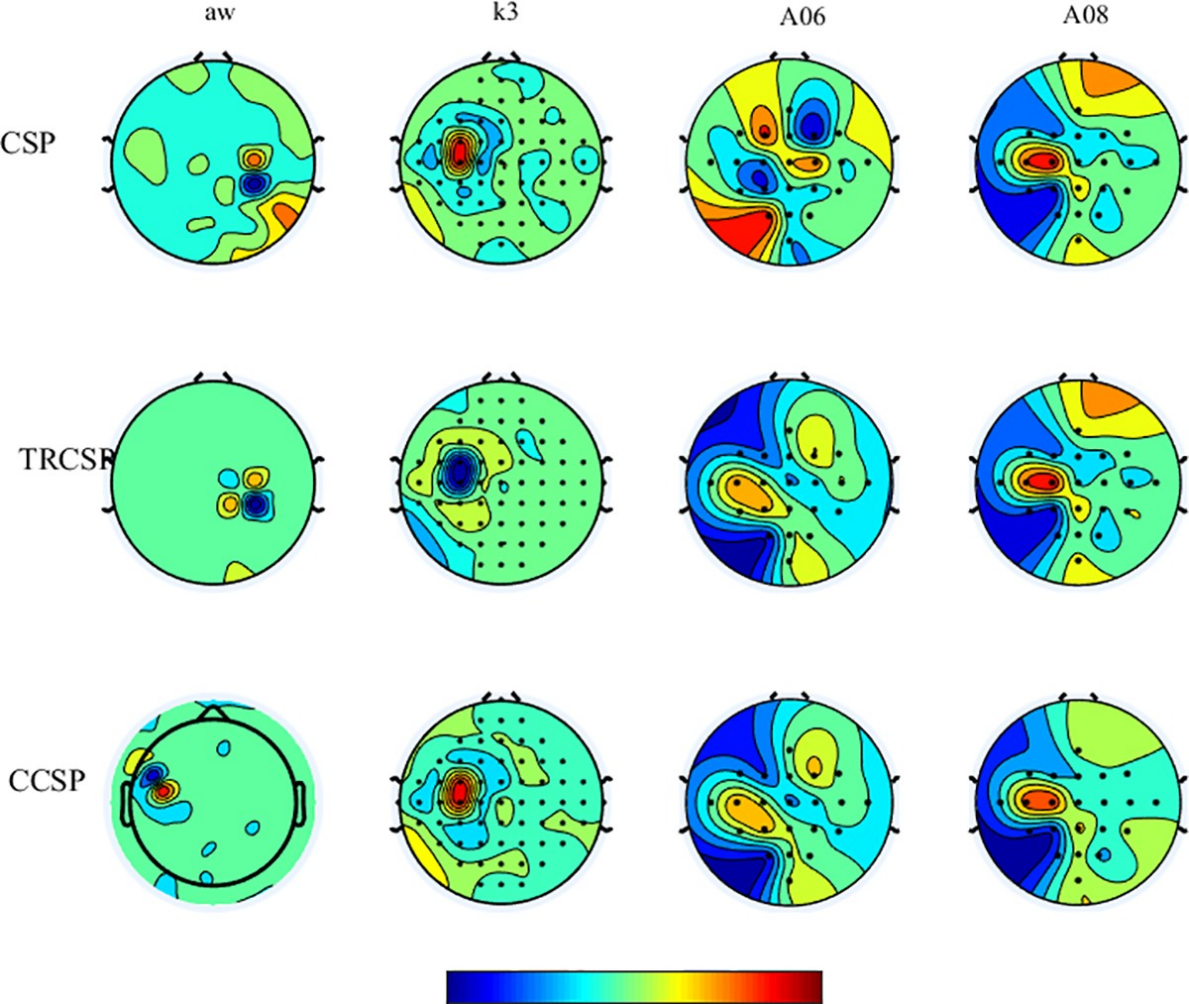
The scalp topography for the first spatial filter using CSP, TRCSP and CCSP methods for the subjects aw, k3, A06 and A08.

After applying spatial filters to the datasets and extracting features from them, they are classified by SVM. In order to test the performance of the CCSP algorithm and compare it with other cases as the benchmark (CSP [7], TRCSP [18], PCA [28] and FDA [37]). The TRCSP algorithm used **K** = **I** as prior information in penalty term **Q**(**w**). The PCA is a mathematical method used for dimension reduction and feature extraction. This technique finds principal components (PCs) to reduce the dimension of data in which the few first components have the largest variances in comparison to the others [28]. FDA is a supervised feature mapping method which is found linear projection so that between-class scatter is maximum; meanwhile within-class scatter is minimum [37]. We used three measures namely classification of accuracy, sensitivity and specificity. Figs 2–6 illustrate the comparison between the classification accuracy of all methods in two-class and four-class scenarios. According to these Figs and regarding the two-class problem, the proposed CCSP algorithm had better performance in 9 out of 17 subjects, while the accuracy results were the same for 4 subjects (k3, al, A03, A08) within all datasets. To explain further, CCSP had better classification accuracy in 9 subjects and same classification accuracy in 4 subjects compared to TRCSP and outperformed CSP in all cases. In addition, CCSP excelled PCA and FDA methods in all cases. The proposed CCSP algorithm had improved classification accuracy rate from 0.63–17.88% compared to the original CSP regardless of the equal cases. This best improvement of classification accuracy occurred in subject aw in the Dataset IVa, BCI competition III which had 90.72%, 88.74%, 72.84%, 64.23% and 52.98% for CCSP, TRCSP, CSP, PCA and FDA respectively. Turning to the four-class problem, CCSP outperformed in 7 out of 12 cases, and it had equal performance in two of them (k3 and A04). In order to clarify more, CCSP had better performance in 8 subjects and had same classification in the rest of them in comparison to CSP. As well as, CCSP outperformed TRCSP in 9 cases but 3 of them. Moreover CCSP surpassed PCA and FDA. Using the proposed approach, the improvement was from 0.69% to 6.67% compared to CSP (regardless of the equal cases). In comparison to CSP, in four-class problem best improvement of classification accuracy belongs to subject k6 in Dataset IIIa, BCI competition III. This subject shows 72.50%, 65%, 65.83%, 25.83% and 49.16% for CCSP, TRCSP, CSP, PCA and FDA respectively in term of classification accuracy. This must be pointed out that the proposed algorithm never had worse performance than the original CSP in all of the subject and two-class and multi-class scenarios. Nevertheless, CSP outperformed TRCSP in 5 subjects. Figs 7 and 8 show boxplots for two-class and multi-class problems, respectively which are shown superiority of CCSP in comparison to the others. In Figs 7 and 8 the distribution of classification accuracy for CCSP is negatively skewed so it shows more than 50% of subjects have classification accuracy more than mean of CCSP method. Maximum and minimum of all of the data are specified in these diagrams. Table 1 reports mean, median and standard deviation of classification accuracy for five methods in two-class and multi-class problems. Compared to the CSP, the proposed method shows about 6.9% and 2.23% improvement in term of mean accuracy in two-class and multi-class problems, respectively. Furthermore, CCSP approach showed about 0.58% and 1.49% improvement in term of mean accuracy in two-class and multi-class problem compared to TRCSP. As well as, in term of mean classification accuracy, the CCSP represents 24.87% and 33.89% improvement in comparison to PCA and 19.23% and 15.43% improvement in comparison to FDA in two-class and multi-class problem respectively. The standard deviation of the CCSP method is better than CSP and TRCSP methods in multi-class problem and has better value than CSP and close to the TRCSP in two-class problem. Table 2 shows sensitivity and specificity for all datasets in two-class problem. Kappa coefficient is statistical method for measuring degree of agreement between classes which is more robust criterion than classification accuracy by considering random agreement. This method assigns zero to the random classification and one to the perfect classification [38]. Then in four-class problem zero is equal to 25% and one is equal to 100% in term of classification accuracy. Table 3 reports kappa coefficient for all datasets in multi-class problem. It shows CCSP outperform other methods in term of kappa coefficient. Best performance for each subject has been reported in boldface in Tables 2 and 3. Table 4 represents running time of five algorithms for all subjects. Running time is the period of all simulation stage including preprocessing, applying spatial filters, feature extraction and classification for testing sets. As it is seen, running time for the proposed CCSP algorithm is approximately as same as the four other approaches. Fig 9 illustrates the first spatial filter using 3 methods for subjects aw (recorded by 118 electrodes), k3 (recorded by 60 electrodes), A06 and A08 (recorded by 22 electrodes) in two-class problem. It can be observed that CSP filter had large electrode weights spread over the unexpected area while CCSP filters provided more emphasis on the electrodes which is located in the motor cortex area [39]. It should be mentioned that the simulation is performed on a computer with 8 gigabytes of RAM and core i7 Intel CPU. Also MATLAB is used as a software in the simulation stage to obtain the results.

**Table 1:** Mean, median and standard deviation (std.) for two and multi-class problem (Best values are in boldface).

		All of the datasets		
		*Mean*	*Median*	*Std*.
Two-class	PCA	58.61%	56.66%	**9.78**
	FDA	64.25	59.82%	15.99
	CSP	76.58%	77.77%	15.62
	TRCSP	82.9%	87.41%	13.43
	CCSP	**83.48%**	**88.07%**	13.71
Multi-class	PCA	37.65%	37.84%	**9.31**
	FDA	56.11	58.64%	12.79
	CSP	69.31%	73.26%	16.22
	TRCSP	70.05%	75.51%	15.42
	CCSP	**71.54%**	**76.07%**	14.93

**Table 2:** Specificity and sensitivity for all of datasets in two-class problem (Best values are in boldface).

		Dataset IIIa, BCI competition III			Dataset IVa, BCI competition III					Dataset IIa, BCI competition IV								
Subject		k3	k6	l1	aa	al	av	aw	ay	A01	A02	A03	A04	A05	A06	A07	A08	A09
Sensitivity	PCA	0.73	**0.86**	0.6	0.56	0.6	0.43	0.4	0.48	0.36	0.7	0.4	0	0.11	0.34	0.11	0.79	0.76
	FDA	0.86	0.7	0.6	0.45	0.92	0.52	0.1	0.57	0.77	0.31	0.72	0.59	0.12	0.59	**0,84**	0.93	0.84
	CSP	**1**	0.76	0.76	0.65	0.96	0.48	0.46	0.81	**1**	**0.62**	0.81	0.48	**0.84**	0.63	0.61	0.95	0.93
	TRCSP	**1**	0.76	0.83	0.8	**1**	0.52	**0.92**	**0.94**	0.94	0.36	**0.93**	0.58	0.7	**0.79**	0.61	**0.97**	0.93
	CCSP	**1**	**0.86**	**0.86**	**0.83**	**1**	**0.53**	0.9	0.92	0.95	0.38	**0.93**	**0.7**	0.51	0.77	0.7	0.95	**0.95**
Specifity	PCA	0.44	0.26	0.53	0.61	0.92	**0.74**	0.88	**0.85**	0.7	0.33	0.66	**1**	**0.87**	0.55	0.83	0.51	0.88
	FDA	0.6	0.36	0.4	**0.76**	0.92	0.58	0.94	0.48	0.66	0.66	0.95	0.62	0.86	0.55	0.45	0.84	**0.91**
	CSP	0.95	0.6	**1**	0.75	**1**	0.52	**0.98**	0.81	0.66	0.5	**1**	0.81	0.16	0.62	**0.94**	0.97	0.88
	TRCSP	**1**	0.93	0.96	0.71	**1**	0.69	0.85	0.8	0.91	**0.83**	**1**	0.9	0.5	**0.65**	0.91	0.97	0.9
	CCSP	**1**	0.73	**1**	0.73	**1**	0.7	0.9	0.84	**0.93**	**0.83**	**1**	0.86	0.59	**0.65**	0.9	**0.98**	0.88

**Table 3:** Kappa coefficient for dataset IIIa, BCI competition III and dataset IIa, BCI competition IV in multi-class problem (Best values are in boldface).

		*Dataset IIIa*, *BCI competition III*			*Dataset IIa*, *BCI competition IV*								
Subject		k3	k6	l1	A01	A02	A03	A04	A05	A06	A07	A08	A09
Kappa coefficient	PCA	0.27	0.01	0.24	0.36	0.05	0.2	0.14	0.004	0.09	0.05	0.19	0.37
	FDA	0.7	0.32	0.41	0.5	0.19	0.48	0.38	0.15	0.16	0.52	0.5	0.62
	CSP	**0.94**	0.54	0.7	0.71	0.3	0.66	**0.55**	0.13	0.29	0.62	0.75	0.77
	TRCSP	0.91	0.53	**0.71**	**0.73**	0.38	0.68	0.54	0.16	0.34	0.65	0.73	**0.79**
	CCSP	**0.94**	**0.63**	0.7	0.72	**0.4**	**0.7**	**0.55**	**0.2**	**0.35**	**0.66**	**0.78**	0.77

**Table 4:** Running time for two-class and multi-class problems.

		time (sec)																
		Dataset IIIa, BCI competition III			Dataset IVa, BCI competition III					Dataset IIa, BCI competition IV								
Subject		k3	k6	l1	aa	al	av	aw	ay	A01	A02	A03	A04	A05	A06	A07	A08	A09
Two-class	PCA	2.11	1.73	2.15	2.57	2.58	2.55	2.39	2.36	3.95	3.01	3.24	3.08	3.10	3.13	3.24	3.49	3.06
	FDA	2	1.75	1.59	1.67	1.71	1.72	1.77	2.21	2.65	2.41	2.47	2.46	2.43	2.39	2.43	2.47	2.52
	CSP	1.86	1.53	1.54	1.81	1.85	1.80	1.78	1.73	2.32	2.47	2.30	2.27	2.31	2.35	2.29	2.35	2.55
	TRCSP	1.91	1.53	1.58	1.84	1.83	1.82	1.76	1.84	2.28	2.32	2.26	2.27	2.33	2.28	2.29	2.29	2.38
	CCSP	1.92	1.57	1.54	1.87	1.81	1.81	1.83	1.82	2.32	2.32	2.28	2.34	2.36	2.28	2.29	2.34	2.31
Multi-class	PCA	2.95	2.51	2.52	-	-	-	-	-	3.12	3.06	3	3.1	3.03	3.04	3.2	3.15	3.15
	FDA	2.9	2.44	2.54	-	-	-	-	-	2.98	2.95	3.07	2.96	2.92	2.96	2.98	2.91	3.06
	CSP	2.17	2.16	2.70	-	-	-	-	-	2.99	2.90	2.84	2.84	2.92	2.78	2.95	2.91	2.83
	TRCSP	2.81	2.20	2.21	-	-	-	-	-	3.02	2.98	2.93	2.99	2.98	2.88	3.01	3.06	2.93
	CCSP	2.87	2.34	2.31	-	-	-	-	-	3.03	3.06	3	2.96	3.09	2.96	3.06	3.06	3.03

## Conclusion

Regularized CSP is used to overcome the shortcomings such as overfitting and noise sensitivity in conventional CSP, by imposing appropriate prior information to its objective function. Several regularization terms are suggested for this purpose. Using prior information in terms of time correlation of the underlying signals can be useful in both two-class and multi-class problems. In this article, we introduced a novel term of prior information to penalize solution of the original CSP, named temporal correlation, which has the advantage of easy extension to multi-class problems. In order to evaluate the performance of the proposed CCSP method, we used 17 subjects in two-class problem and 12 subjects in multi-class problem. We further compared its simulation results with the classical CSP, TRCSP, PCA and FDA. In two-class problem, the results show that our approach outperforms CSP, TRCSP, PCA and FDA by 6.9%, 0.58%, 24.87% and 19.23% in the mean of classification accuracy, respectively. Likewise, in multi-class scenario, the proposed CCSP achieves an improvement of 2.23%, 1.49%, 33.89% and 15.43% in classification accuracy mean in comparison to the CSP, TRCSP, PCA and FDA methods. Also CCSP method provided neurophysiological plausibility spatial filters. We can suggest using the proposed algorithm as a processing unit in the MI-based BCI systems to have better recognizing between the MI signals. It should be noticed we did not investigate the performance of proposed method in datasets with small training data. As well as when EEG has been recorded with few numbers of electrodes our algorithms need to more investigation. As well as the proposed method needs more calculation to obtain optimized spatial filters therefore in the systems with weak hardware it maybe takes time to operate.
