# Correction: Correlation-based common spatial pattern (CCSP): A novel extension of CSP for classification of motor imagery signal

**DOI:** 10.1371/journal.pone.0303765

**Published:** 2024-05-09

**Authors:** Khatereh Darvish ghanbar, Tohid Yousefi Rezaii, Ali Farzamnia, Ismail Saad

The corresponding author’s email address is incorrect. Ali Farzamnia’s email address is: alifarzamnia@ums.edu.my.
